# High-performance flexible energy storage and harvesting system for wearable electronics

**DOI:** 10.1038/srep26122

**Published:** 2016-05-17

**Authors:** Aminy E. Ostfeld, Abhinav M. Gaikwad, Yasser Khan, Ana C. Arias

**Affiliations:** 1 Department of Electrical Engineering and Computer Sciences, University of California, Berkeley, California 94720, USA

## Abstract

This paper reports on the design and operation of a flexible power source integrating a lithium ion battery and amorphous silicon solar module, optimized to supply power to a wearable health monitoring device. The battery consists of printed anode and cathode layers based on graphite and lithium cobalt oxide, respectively, on thin flexible current collectors. It displays energy density of 6.98 mWh/cm^2^ and demonstrates capacity retention of 90% at 3C discharge rate and ~99% under 100 charge/discharge cycles and 600 cycles of mechanical flexing. A solar module with appropriate voltage and dimensions is used to charge the battery under both full sun and indoor illumination conditions, and the addition of the solar module is shown to extend the battery lifetime between charging cycles while powering a load. Furthermore, we show that by selecting the appropriate load duty cycle, the average load current can be matched to the solar module current and the battery can be maintained at a constant state of charge. Finally, the battery is used to power a pulse oximeter, demonstrating its effectiveness as a power source for wearable medical devices.

The number and variety of electronic devices has dramatically increased in the past 5 years and currently there is growing interest in electronic devices with flexible, thin, and large-area form factors. These electronics span a vast range of applications including mobile devices^1^, healthcare^2^,^3^, smart surfaces^4^, smart packaging^5^, and wearables such as smart watches and e-textiles^6^,^7^,^8^. All of these diverse applications require electrical power, and many of them, especially wireless communication and light-emitting devices, require relatively large current pulses on the order of many milliamps. For example, the reported peak current consumption of Bluetooth Low Energy wireless communication in a wearable sensor module was 18 mA^9^, and a smart watch such as the Samsung Gear 2 consumes up to 48 mA during calls^7^. For most mobile devices today this power is provided by a battery that is designed to be recharged each night using a wired connection. However, as the number of devices grows, so does the need for power sources that can meet their energy demands without frequent wired charging cycles. Minimizing wired charging is particularly important for wireless sensors as well as for health monitoring devices, where the consequences of a user forgetting to plug in the device can be severe.

For such applications it is crucial to have a battery with high areal capacity, so that it can store a large amount of energy without being rigid and bulky. Discharge at a high rate is also needed to support the peak current consumption of the load device. Charging would become more convenient if the battery is combined with one or more devices that harvest energy from ambient sources, such as light, thermal, or vibrational energy^4^,^10^,^11^,^12^,^13^. Furthermore, taking advantage of the many recent advances in flexible electronics technology, the energy harvester, battery, and load devices should be physically integrated into a single user-friendly flexible package. To date, several flexible thin-film rechargeable battery chemistries and architectures^9^,^14^,^15^,^16^,^17^,^18^ and energy harvesting technologies^19^,^20^,^21^,^22^ have been reported. However, an effective energy harvesting and storage system requires not only high-performing individual components, but also good compatibility between components. When designing such a system, it is necessary to consider both average and peak power consumption of the load, as well as the power density that can be harvested from the available ambient energy sources. The type and dimensions of the battery and energy harvester can then be selected in order to power the load efficiently for the desired length of time before wired charging is required.

Designing a battery that is mechanically flexible and can also provide the required capacity and discharge rate for wearable and wireless electronics is extremely challenging. Lithium ion batteries have been the choice of battery chemistry for powering consumer electronics due to their high energy and power density, and stable electrochemical performance^23^,^24^. Traditionally, lithium ion batteries use a “Jelly Roll” architecture, where the anode and cathode are stacked together with a polymeric separator, rolled, and encapsulated within metal-laminated pouches or rigid plastic containers under compression. Batteries with “Jelly Roll” architecture have high capacity (mAh) and energy density (Wh/L), but they are bulky and non-flexible. For the fabrication of flexible batteries, a thin film configuration is adopted where single layers of anode, separator and cathode are stacked together and sealed within a flexible encapsulation. Parameters such as mechanical properties of the current collector, thickness of the active layers, choice of polymeric binder and the encapsulation material are important factors in the design of a flexible battery. In the past few years, a number of innovative designs have been demonstrated for the fabrication of flexible and stretchable batteries. Current collectors based on carbon nanotubes^25^,^26^, conductive fabrics^27^,^28^, conductive ink^29^ and carbon foams^30^ have been used to improve the flexibility of the electrodes and adhesion to the active layers. The mechanical integrity of the active layers has also been improved by supporting the active particles within porous membranes^31^,^32^, carbon nanotubes^33^,^34^ and graphene networks^30^ or by synthesizing the active materials directly on conductive supports^35^. Such designs have greatly improved the flexibility and mechanical properties of thin film batteries. However, even with design advances, the areal capacity (mAh/cm^2^) and rate capabilities of flexible batteries have been poor. Due to low thickness of the active layers, the areal capacities have been in the range of 0.1 to 0.5 mAh/cm^2^ 27,36,37. In addition, the low conductivity of flexible current collectors and their poor electrical contact to the active layers have limited the maximum operating discharge rate to less than C/2 rate^7^,^26^,^27^,^38^.

Of the various energy harvesting methods, photovoltaics (PV) are often the best suited for charging high-capacity batteries because of the relatively high available power density on the order of 100 mW/cm^2^ outdoors or hundreds of μW/cm^2^ indoors, compared to tens to hundreds of μW/cm^2^ for thermal and vibrational sources^4^,^13^. Other energy harvesting methods such as triboelectric generators have been developed recently^39^, but the low charging rate limits these devices to relatively low-capacity batteries. The selection of an appropriate PV module for a particular battery and application depends on the expected illumination conditions, as irradiance can vary by a factor of 100 or more from typical indoor lighting to sunny outdoor conditions^40^. The short-circuit current of a PV module is roughly proportional to the irradiance of the incident light, while the open-circuit voltage is only logarithmically dependent on the irradiance^41^. Ideally, the PV module should be able to provide power over the entire range of battery voltages and expected illumination conditions; in other words, the open-circuit voltage of the PV module should be at least equal to the maximum battery voltage. For most efficient utilization of the PV module, the maximum power point voltage of the PV module should be within the voltage range of the battery. A large-area PV module is desirable to provide a high charging current under low irradiance conditions. However, if the area of the module is too large, the charging current under high irradiance may induce a high potential drop in the battery and the battery would reach its upper voltage limit before being fully charged. To date there have been several demonstrations of flexible PV modules charging both commercial^42^,^43^ and custom^27^,^37^,^44^ flexible batteries, but the performance often suffers from limited battery lifetime^37^,^44^ or low battery capacity at high charging rates^27^,^37^.

Here we consider the pulse oximeter as an example wearable electronic load and design a flexible high-performance energy harvesting and storage system to meet its power requirements. The pulse oximeter monitors a patient’s heart rate and blood oxygenation by measuring the light absorbed in the blood at two different wavelengths. Due to the high power consumption of the optoelectronic probe, the pulse oximeter requires a high current of 20 mA during each measurement, compared to 1 mA between measurements. The lifetime of the battery between charging events will depend on the oximeter’s duty cycle—the fraction of the time that it is measuring—in addition to the battery capacity and the available power from energy harvesters. Thus, the design of a reliable power supply for this application requires a battery with high capacity and rate capability, an energy harvester with relatively high power density (such as PV) and appropriate voltage to charge the battery, and the selection of an appropriate duty cycle for the oximeter in order to match the average power generated and consumed. A solar powered pulse oximeter has recently been demonstrated using rigid components^45^, but since the optoelectronic probe must be in close contact with the skin, making the components including the power supply flexible could greatly improve user comfort. Flexibility also improves signal integrity, since wrapping the device around a patient’s finger reduces the parasitic current due to ambient light^2^. Ideally, the pulse oximeter system would be constructed as a single flexible piece as shown in Fig. 1a, comprising the optoelectronic probe, data processing electronics, and a flexible power source consisting of layered battery and PV module. In this work, a flexible thin-film lithium ion battery was designed for this application and integrated with a flexible thin-film amorphous silicon photovoltaic module. This energy harvesting and storage system is shown schematically in Fig. 1b and a photograph is given in Fig. 1c. Since both PV module and battery are flexible, the entire system can also be flexed and attached to flexible or curved surfaces, as shown in Fig. 1d–g. Detailed characterization of the battery was performed prior to its integration with the PV module or loads. Then, the behavior of the energy system was characterized under multiple lighting conditions and load duty cycles, and its ability to power the pulse oximeter was verified.

## Results and Discussion

### Flexible lithium ion batteries

**Figure 2:**
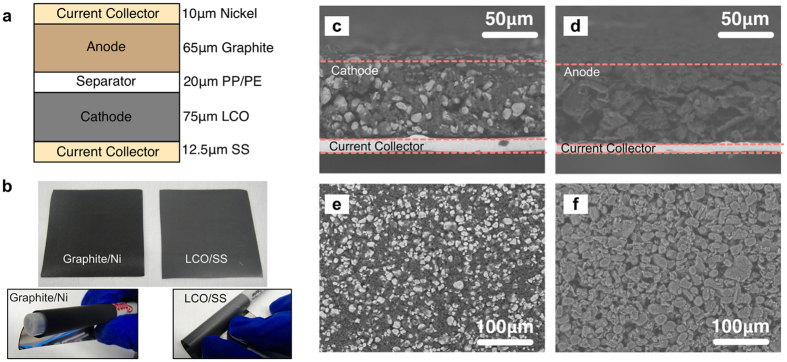
(**a**) Cross-sectional schematic of the lithium ion battery. (**b**) Optical image of graphite electrode on nickel foil and LCO electrode on stainless steel, flat (top) and flexed over a pen with diameter of 10 mm (bottom). Cross-sectional SEM micrographs of LCO (**c**), and graphite (**d**) electrodes, respectively. Topographical SEM micrographs of LCO (**e**), and graphite (**f**) electrodes, respectively.

The current collector of a battery provides mechanical support to the active layers and helps with efficient charge insertion and removal from the battery. Here, stainless steel (12.5 μm) and nickel (10 μm) foils were found to be suitable current collectors for the cathode and anode, respectively. These foils are compliant and highly conductive, remaining mechanically robust even after repeated flexing. Lithium cobalt oxide (LCO) and synthetic graphite were selected as the active materials for the cathode and anode, respectively, due to their stable electrochemical performance. The cross-sectional schematic of the battery is shown in Fig. 2a. To form the active layers, the active particles are mixed with a conductive additive to increase the electronic conductivity and a polymeric binder to hold the particles together. The active particles and conductive additives are inherently rigid and non-flexible, but when mixed with a polymeric binder, deposited in the form of slurry and dried, a porous structure is formed. This porous structure can absorb compressive and tensile stresses generated in the electrodes during flexing. Figure 2b shows optical images of the flexible graphite and LCO electrodes (4 inch^2^ each) flat and flexed over a pen with diameter of 10 mm. Figure 2c–f show the cross-sectional and topographical scanning electron microscope (SEM) micrographs of the LCO and graphite electrodes. The thickness of the LCO and graphite electrodes after printing and calendaring was 75 and 65 μm, respectively. The SEM images show that the active materials and conductive additives are well distributed and adhere well to the current collector foils. The battery had a theoretical areal capacity of ~2 mAh/cm^2^ and thickness of 182.5 μm. After encapsulation within aluminum-laminated pouches, the thickness of the battery increased to 420 μm, although this can be reduced by engineering low-thickness encapsulation^46^.

**Figure 3:**
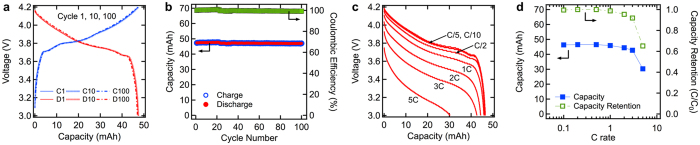
(**a**) 1st, 10th and 100th charge-discharge curves of the battery cycled between 4.2 and 3.0 V at C/5 rate. (**b**) Charge-discharge capacity (mAh) and coulombic efficiency of the battery cycled for 100 cycles at C/5 rate. (**c**) Discharges curves of the battery discharged at C/10, C/5, C/2, 1C, 2C, 3C and 5C rate. (**d**) Discharge capacity (mAh) and capacity retention of the battery discharged at C/10, C/5, C/2, 1C, 2C, 3C and 5C rate.

The lithium ion battery was cycled for 100 cycles at C/5 rate between 3.0 and 4.2 V. Figure 3a shows the 1^st^, 10^th^ and 100^th^ charge-discharge curves of the battery, which lay on top of each other, demonstrating excellent electrochemical stability^47^. Figure 3b shows the charge-discharge capacity and the coulombic efficiency of the battery during this period. The battery had capacity of 47.5 mAh (1.84 mAh/cm^2^), energy density of 383 Wh/L without the encapsulation (167 Wh/L with encapsulation), and areal energy density of 6.98 mWh/cm^2^. The battery was able to retain 99.2% of its initial capacity after 100 cycles, and the coulombic efficiency of the battery was above 99.9%. The rate capability was studied by discharging the battery at rates of C/10 to 5C between 4.2 and 3.0 V. Between discharging cycles the battery was charged using a CC-CV (constant current–constant voltage) charging procedure, during which time it was charged at C/4 rate until it reached 4.2 V and then held at 4.2 V for 1 hour to ensure complete charging. The current dropped to less than 0.1 mA at the end of 1 hour. Figure 3c shows the discharge curves of the battery from C/10 to 5C discharge rate. There was negligible drop in capacity below 1C discharge rate, and the battery was able to provide 93% of its theoretical capacity at 3C rate. The voltage drop at the start of discharge at high C rates is due to ohmic losses in the battery, which include contact losses between the contact tabs and current collector and between the current collector and active layer, as well as particle-to-particle contact resistance and conductivity of the electrolyte. Figure 3d shows the capacity (mAh) and capacity retention (C/C_0_) of the battery discharged at C/10 to 5C rate. The capacity retention of the battery at high C rates is considerably better than previous reports on flexible batteries with similar electrode design^7^,^27^,^37^,^38^. In most previous flexible battery designs, a combination of low conductivity of the current collectors and poor electrical contact between the active layer and current collectors led to high potential drops when the battery was operated above C/2 rate.

**Figure 4:**
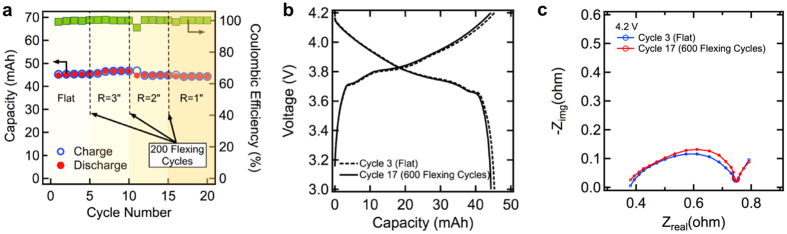
(**a**) Charge-discharge capacity of the battery electrochemically cycled at C/5 rate under flat state and after flexing 200 times to 3, 2 and 1 inch bending radius. (**b**) Charge-discharge curves of the battery under flat state and after bending for 600 cycles. (**c**) ElS curves of the battery under flat state and after bending for 600 times.

The effect of mechanical flexing on the electrochemical performance of the battery was studied by cycling the battery for 20 electrochemical cycles at C/5 rate; at the end of the 5^th^, 10^th^ and 15^th^ electrochemical cycles the battery was flexed 200 times to a bending radius of 3, 2 and 1 inch respectively. Flexing can degrade the performance of the battery by two processes: swelling and delamination, which depend on the electrode design, size of the particles, nature of the binder, bending radius during flexing, and speed of flexing. Repeated flexing leads to swelling of the active layer, which increases the impedance within the electrodes due to loss of particle-to-particle contact. Delamination of the active layer will occur if the shear stress at the contact between the current collector and the active layer is higher than the peel strength. Figure 4a shows the charge-discharge capacity and coulombic efficiency of the battery under flat state and after each period of flexing. The capacity retention for the battery after 20 electrochemical cycles and a total of 600 flexing cycles was 98.8%. Figure 4b shows the charge and discharge curves of the unflexed battery (cycle 3), and after flexing the battery 600 times (cycle 17). A slight drop in capacity can be related to an increase in the impedance within the battery. Electrochemical impedance spectroscopy (EIS) was carried out before and after flexing the battery to understand the effect of flexing on the mechanical integrity of the battery^26^,^48^. Figure 4c compares the EIS data before and after flexing the battery 600 times. The intercept at high frequency represents the resistance of the electrolyte between the current collector foils. The first semicircle loop represents the total contact resistance, which is a combination of contact resistances between the current collector and active layer, particle-to-particle contact and passivation layer. For LCO-graphite based full cells, the contact resistance between the current collector and active layer dominates the resistance in the first loop. The second semicircle loop represents the charge transfer resistance for the electrochemical reaction, and the straight line at low frequency is due to solid-state diffusion of lithium ions with the electrolyte. The size of the first semicircle remains constant after 600 flexing cycles, indicating that the mechanical integrity of the battery is maintained after flexing. Furthermore, SEM micrographs of the battery electrodes after 100 charge/discharge cycles and 600 flexing cycles, given in Supplementary Fig. S1, show no sign of delamination. The increase in diameter of the second semicircle is related to the ageing of the electrodes^26^.

Compared to recent research reports on flexible batteries using novel materials and structures, the areal energy density and volumetric energy density of our battery are considerably higher, as shown in Supplementary Fig. S2 and Table S1^27^,^36^,^49^,^50^,^51^,^52^,^53^. In most of these flexible batteries, the loading of the active materials is kept low to improve the flexibility of the electrodes, which reduces the energy density. For the purpose of powering a wearable device, the battery should be flexible, but the capacity should also be high to ensure that the battery can power the device for a reasonable period of time. Thus, high volumetric energy density is important to achieve the desired capacity while keeping the volume of the battery small. By using low thickness metal current collectors (10–12.5 μm) and separator (20 μm), and relatively thick active layers (65–75 μm), we were able to fabricate a flexible battery with a high areal and volumetric energy density. The energy density of flexible batteries can be further increased by reducing the thickness of inactive layers in the battery, and by increasing the thickness of active layers while ensuring that the maximum strain experienced by the electrodes during flexing never reaches its critical limit.

### Battery charging with photovoltaic module

**Figure 5:**
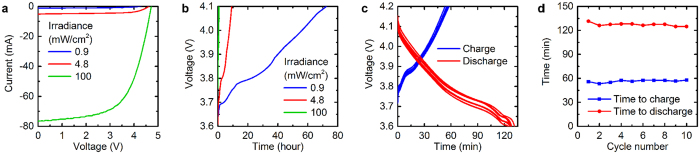
Electrical characteristics of flexible PV module charging the flexible battery. (**a**) Current-voltage characteristics of the PV module and (**b**) battery voltage over time as it is charged by the PV module, under different illumination conditions. (**c**) Battery voltage profiles over 10 charge/discharge cycles. Charging is performed using the PV module under 100 mW/cm^2^ irradiance and discharging is performed at a constant current of 20 mA. (**d**) Time for the battery to charge to 4.2 V and discharge to 3.6 V over the same 10 cycles as in (**c**).

To create an energy storage and harvesting system, the flexible lithium ion battery was combined with a flexible amorphous silicon PV module having similar dimensions and compatible voltage. The current-voltage characteristics of the PV module are shown in Fig. 5a under three lighting conditions: a compact fluorescent light bulb at two different heights to produce irradiance of 0.9 and 4.8 mW/cm^2^, and simulated solar illumination of 100 mW/cm^2^. As expected, the current of the PV module was strongly dependent on the irradiance, while the voltage was only slightly affected; the maximum power point voltage was in the 3–4 V range for all three illumination conditions. The battery was charged from 3.6 V to 4.1 V by the PV module under each illumination condition, as shown in Fig. 5b. As expected, the time for the battery to charge was inversely proportional to the average PV module current. When the irradiance was increased from 0.9 to 4.8 mW/cm^2^, the average PV module current over the 3.6–4.1 V range increased from 0.46 mA to 3.9 mA, a factor of about 8. The time for the battery to charge correspondingly decreased by a factor of 8, from 72 hours to 9.2 hours. Under 100 mW/cm^2^, the higher PV module current introduced a noticeable potential drop in the battery, increasing the voltage at the start of charging to 3.75 V. The average PV current over this charging voltage range was 51 mA, 12 times higher than the current in the 4.8 mW/cm^2^ condition, and the battery charge time was correspondingly 12 times lower, 0.77 hours. This range of light intensities corresponds to charging rates varying from C/72 to greater than 1C. Under all of the illumination conditions, the charge supplied to the battery over the 3.6- 4.1 V range was calculated to be approximately 40 mAh, which is consistent with the capacity over that voltage range presented in Fig. 3a. Supplementary Fig. S3 shows current-voltage characteristics and a charging curve with a blocking diode, indicating the effectiveness of the diode at allowing forward current from the PV module while blocking reverse current.

The battery was shown previously in Fig. 3b to have excellent lifetime when cycled at C/5 rate. However, the battery would be subjected to a much higher charging rate under sunlight and higher discharging rates when powering electronic devices such as the pulse oximeter. To determine the suitability of the battery for these applications, we assessed the stability of the battery as it was repeatedly charged to 4.2 V using the PV module under full sun (100 mW/cm^2^) and discharged to 3.6 V at a rate of 20 mA. The battery voltage profile during each charge and discharge cycle is plotted in Fig. 5c, for 10 cycles. Figure 5d plots the time required for the battery to reach 4.2 V while charging and 3.6 V while discharging, showing that the performance of the battery was relatively stable over the 10 cycles. The variation in the charging and discharging time may be attributed to variation in temperature of the battery and PV module, and the resulting variation in PV module voltage and battery potential drop.

### Battery and photovoltaic module powering loads

The battery was employed to power a pulse oximeter, consisting of optoelectronic probe, light-emitting diode (LED) driver, photodiode read circuit, and microcontroller. For the purposes of activity tracking, the pulse oximeter turns on periodically to collect pulse and blood oxygenation measurements. During operation, the optoelectronic probe consumes around 16 mA of current and the data acquisition and processing units consume around 4 mA. In the “off” state, the oximeter system turns off the optoelectronic probe and the data acquisition module; however, the microcontroller and the serial communication module stay on, consuming around 1 mA of current. The lifetime of a battery powering the oximeter depends on the duty cycle, the fraction of the time the oximeter is in the “on” state; an appropriate power source should be able to power the oximeter for many hours before charging is required. We first used an electronic load alternating between 20 mA and 1 mA with variable duty cycle, simulating the pulse oximeter alternating between its “on” and “off” states, to characterize the lifetime of the energy storage and harvesting system under different illumination conditions.

**Figure 6:**
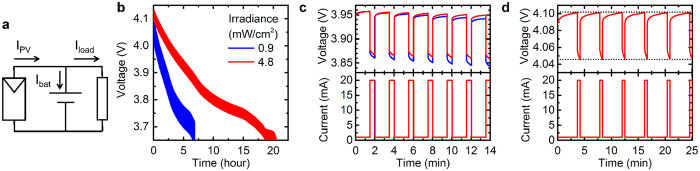
Behavior of PV module, battery and load connected together. (**a**) Circuit schematic of PV module, battery and load, indicating currents flowing in each component. (**b**) Discharge curves of the battery with two PV module irradiance conditions. The load alternates between 20 mA for 30 seconds and 1 mA for 90 seconds. (**c**) Closeup of the battery voltage and load current waveforms shown in (**b**) over a few load cycles. (**d**) Battery voltage and load current waveforms with the load alternating between 20 mA for 30 seconds and 1 mA for 220 seconds. The dotted black lines are a guide to the eye indicating that the battery voltage at the end of each cycle does not change.

When a battery is connected to a photovoltaic module and load simultaneously, the additional energy collected by the PV module slows the rate of discharge of the battery, prolonging the time the battery can operate before it must be charged. To characterize the system behavior under these conditions, a PV module, battery and load were connected in parallel as shown in Fig. 6a. The current into the battery in this configuration, *I*_*bat*_, is equal to the difference between PV and load currents, *I*_*PV*_ *–* *I*_*load*_; the sign of this value determines whether the battery will charge or discharge. Here, the “on” state lasted for 30 seconds and the “off” state for 90 seconds, giving a duty cycle of 25% and an average load current of 5.75 mA. For both the indoor illumination conditions, 0.9 and 4.8 mW/cm^2^, the current produced by the PV module is less than 5.75 mA, so the battery would be expected to discharge overall. Specifically, with PV module currents of 0.46 and 3.9 mA, respectively, the battery should discharge at average currents of 5.3 and 1.8 mA. The voltage was monitored with both illumination conditions as the battery discharged, as shown in Fig. 6b,c. Figure 6b shows the full discharge curves, while Fig. 6c shows a closeup of a few load cycles, allowing the voltage and current waveforms to be distinguished. The difference between the voltage under 20 mA load and under 1 mA load is due to the higher potential drop of the battery under the higher discharge rate. Increasing the irradiance from 0.9 to 4.8 mW/cm^2^ clearly increases the lifetime of the battery. For example, under 0.9 mW/cm^2^ it took 4.8 hours for the voltage in the 20 mA load phase to decrease from 4.0 V to 3.7 V. Under 4.8 mW/cm^2^, this same decrease occurred over 15 hours, 3 times longer. This is consistent with the threefold reduction in the calculated average discharge current. The slower discharge under higher irradiance is visible after just a few cycles in Fig. 6c.

While increasing the irradiance has been proven effective at slowing the discharge of the battery, in some applications the battery may be required to last even longer between charging, or it may not be feasible for the user of the device to adjust the illumination. In these cases, it may be preferable to adjust the load duty cycle instead. If the average load current matches the PV module current exactly, the battery lifetime can in theory be extended indefinitely. For example, starting with a charged battery with voltage of 4.1 V, we found the PV module current under 4.8 mW/cm^2^ at this voltage to be 3.4 mA. If the time in the “on” state remains at 30 seconds, the load should be off for 210 seconds to give an average current of 3.4 mA. It was determined experimentally that a 220-second “off” state or duty cycle of 12% resulted in no net charge or discharge of the battery, which is in good agreement with the calculated value. Figure 6d shows the battery voltage measured over a few load cycles with these conditions, indicating that the battery voltage (and therefore also its state of charge) is the same after each load cycle. If the load contains devices such as microcontrollers that perform computation, it may be possible to monitor the battery voltage and adjust the duty cycle in real time as illumination conditions change, to ensure the battery remains charged over extended periods of time.

**Figure 7:**
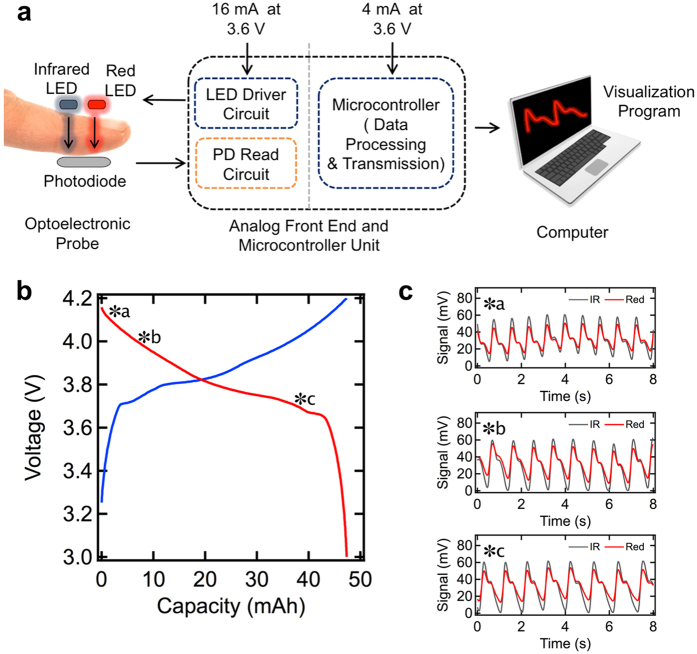
(**a**) Diagram of the system for obtaining photoplethysmogram (PPG) signals from pulse oximeter. (**b**) Charge-discharge characteristic of the battery, highlighting three voltages (*a, *b, *c) at which the battery was used to power the pulse oximeter. (**c**) PPG signals obtained at the three battery voltages.

Finally, pulse oximetry measurements were collected using the flexible battery as the power source. For greatest utilization of the battery capacity, the pulse oximeter should be able to function over a range of voltages. To assess the effect of battery voltage on the performance of the pulse oximeter, the PPG signal of a volunteer’s index finger was collected as shown in Fig. 7a using a battery at varying states of charge. Figure 7b reviews the charge and discharge curves of the battery and highlights three points at which measurements were taken. The PPG signals at each of those points are shown in Fig. 7c, indicating that a high quality signal can indeed be obtained over a range of battery voltages and states of charge.

## Conclusion

In summary, we have developed a flexible power source integrating a high-performance thin-film lithium ion battery with an amorphous silicon photovoltaic module, designed for wearable electronics applications. Both battery design and overall system design are crucial in order to achieve robust and effective power sources with minimal charging requirements. We emphasize the importance of both levels of design by demonstrating a battery with high areal capacity, rate capability, and flexibility, as well as analyzing the relationships between irradiance, load current profile, and battery lifetime. To optimize the battery, thin nickel and stainless steel foils were utilized as current collectors, and LCO and graphite electrodes were fabricated with porous structures. As a result, the battery was stable throughout charging and discharging at rates up to 3C under repeated charge/discharge cycling and flexing. We then demonstrated charging of the battery from the PV module with irradiance ranging from 0.9 to 100 mW/cm^2^, representing both indoor and outdoor illumination conditions. Supplementing the battery with additional photovoltaic power was shown to increase the length of time the battery could power a load before becoming fully discharged. Optimizing the load duty cycle to balance the average load current and the photovoltaic module current allowed the battery to be maintained at a constant state of charge. The battery was also used to power a pulse oximeter, confirming its suitability as a power supply for wearable health monitoring devices. Although this work focuses specifically on photovoltaic energy harvesting and the pulse oximeter, the process of matching the average currents can be extended to other types of energy harvesters and electronic loads. Similarly, the advances in flexible battery technology we present here can help the vision of ubiquitous, flexible, wireless electronics to be realized.

## Methods

### Batteries

Lithium cobalt oxide (LCO) and synthetic graphite were used as the active materials for the cathode and anode, respectively. The cathode slurry was a mixture of 80 wt% LCO (MTI Corp.), 7.5 wt% graphite (SFG 6L, TIMCAL), 2.5 wt% carbon black (Super C65, TIMCAL) and 10 wt% polyvinylidene fluoride (PVDF, Kureha Corp.) in N-methyl-2-pyrrolidene (NMP, Sigma Aldrich) as the solvent. The anode slurry was a mixture of 90 wt% synthetic graphite (MTI Corp.), 4.5 wt% carbon black (Super C65, TIMCAL) and 5.5 wt% PSBR (Targray Technology) in deionized water as the solvent. The slurries were homogenized by stirring overnight using a vortex mixer. The cathode and anode slurries were printed over stainless steel (SS, 12.5 μm, Grainger) and nickel (10 μm, Targray Technology) foils, respectively, using a doctor blade, at a speed of 15 mm/s. The electrodes were heated in an oven at 80 °C for two hours. The thickness of the LCO and graphite electrode after calendaring to a porosity of 30% was 75 and 65 μm, respectively. The areal loadings of the cathode and anode were 17.85 and 7.17 mg/cm^2^, respectively. The electrodes were cut to a dimension of 2 × 2 inch^2^ (25.81 cm^2^). The theoretical capacities of the cathode and anode were 53.8 mAh (2.09 mAh/cm^2^) and 58.23 mAh (2.26 mAh/cm^2^), respectively (theoretical specific capacities of LCO and graphite are 145 and 350 mAh/g, respectively). The cathode was the limiting electrode in the battery. The ratio of the theoretical capacity of the anode to cathode was 1.08.

Before assembling the battery in the glove box, the electrodes were heated overnight in a vacuum oven connected to the glove box at 140 °C for 12 hours to remove traces of residual solvent from the electrodes. Full cells were prepared by stacking the anode (graphite on Ni foil) and cathode (LCO on SS foil) with a polypropylene based separator (20 μm, Celgard). The stack was soaked with an electrolyte solution of 1M LiPF_6_ in EC/DEC (1:1) (MTI Corp.). Nickel and aluminum tabs were used to make electrical contact to the current collectors of the anode and cathode, respectively. The stack was heat sealed within an aluminum-laminated pouch (polyethylene/aluminum/polypropylene, Sigma Aldrich). After sealing, the battery was allowed to rest for a day to ensure complete wetting of the electrodes.

The batteries were electrochemically cycled using a battery analyzer (MTI Corp.). During the formation cycles, the battery was cycled at C/20 rate (2.69 mA, based on theoretical capacity of the cathode) between 4.2 and 3.0 V for three cycles. The coulombic efficiency in the first cycle was around 85 to 88% due to the consumption of lithium ions during the formation of the solid electrolyte interface (SEI) layer, but increased to above 99.9% the end of three cycles. The capacity of the battery after the formation cycles was 47.5 mAh. The C-rate of the battery for further electrochemical experiments was based on the discharged capacity accessed at the end of the third formation cycle, i.e. 1C = 47.5 mA. Electrochemical impedance spectroscopy (EIS) scans were carried out using a potentiostat (Ivium) at frequencies ranging from 10^6^ to 0.1 Hz and at amplitude of 10 mV. The SEM micrograph images were taken using a tabletop SEM (Hitachi). The batteries were flexed by bending them over plastic tubes of radii ranging from 3 to 1 inch.

### Energy system

Flexible amorphous silicon photovoltaic modules (Powerfilm MPT3.6–75) were used to charge the batteries. The PV module current-voltage characteristics and battery charging characteristics were obtained using a Keithley 2400 source-meter under conditions representing both indoor and outdoor illumination. The “outdoor” condition, air mass (AM) 1.5 global illumination with irradiance of 100 mW/cm^2^, was simulated with an Oriel Sol1A solar simulator. Two indoor conditions were compared, using a 13 W compact fluorescent light bulb at two different distances from the PV module, 5 cm and 16 cm. Irradiance was approximately 4.8 and 0.9 mW/cm^2^ respectively for the two distances, as measured using a photodiode (Hamamatsu 66R) and spectrometer (Thorlabs CCS 200). A flexible power supply integrating a PV module, battery and blocking diode (Schottky diode, ON Semiconductor MBRM130LT3G) was also constructed. The electrical connections were made using a combination of copper foil, conductive epoxy, and soldering.

To characterize the behavior of the battery with both solar module and load connected simultaneously, the Keithley source-meter was configured to repeatedly draw 20 mA for a specified time, followed by 1 mA for a specified time, simulating the power consumption of the pulse oximeter during its “on” and “off” states. The PV module was exposed to either 4.8 or 0.9 mW/cm^2^ of light using the compact fluorescent bulb as before. The source-meter monitored the load current and voltage throughout the process.

A pulse oximeter based on a Texas Instruments MSP430 microcontroller was used for the oximeter data acquisition and processing. A photoplethysmogram (PPG) signal was acquired from a volunteer’s index finger using red (632 nm) and infrared (940 nm) light-emitting diodes (LEDs) and a silicon photodiode (PD) at 1 kHz. The PD current signal was then filtered, amplified and converted to a voltage signal. A 10-bit analog to digital converter (ADC) was used to digitize the analog signal. Finally, universal asynchronous receiver/transmitter (UART) protocol was used to send the processed data to a computer for visualization. The optoelectronic probe, LED driver, PD read circuit, and microcontroller board were powered using a flexible battery.

## Additional Information

**How to cite this article**: Ostfeld, A. E. *et al*. High-performance flexible energy storage and harvesting system for wearable electronics. *Sci. Rep*. **6**, 26122; doi: 10.1038/srep26122 (2016).

## Supplementary Material

Supplementary Information

## Figures and Tables

**Figure 1:**
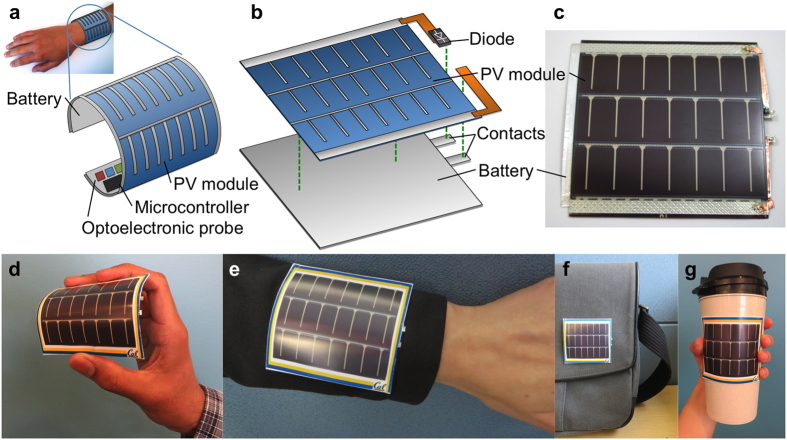
(**a**) Illustration of activity-tracking wristband concept containing flexible battery, PV energy harvesting module, and pulse oximeter components. (**b**) Diagram and (**c**) photograph of a flexible energy harvesting and storage system comprising PV module, battery, and surface-mount Schottky diode, showing the components and attachment points. The diode is included to prevent discharge of the battery into the PV module in low-light conditions. (**d–g**) Photographs of the device being flexed in the hand (**d**) and on various flexible and curved surfaces: jacket sleeve (**e**), bag (**f**), and travel mug (**g**).
