# Supplementary material for: High-performance flexible energy storage and harvesting system for wearable electronics
Source: Sci Rep. 2016 May 17;6:26122. doi: 10.1038/srep26122 (PMC4869018; doi:10.1038/srep26122)
Supplement: Supplementary Information [file srep26122-s1.pdf]

Supplementary Information

High-performance flexible energy storage and harvesting system for wearable electronics

Aminy E. Ostfeld,‡ Abhinav M. Gaikwad,‡ Yasser Khan, and Ana C. Arias

Department of Electrical Engineering and Computer Sciences, University of California, Berkeley, CA 94720, USA.

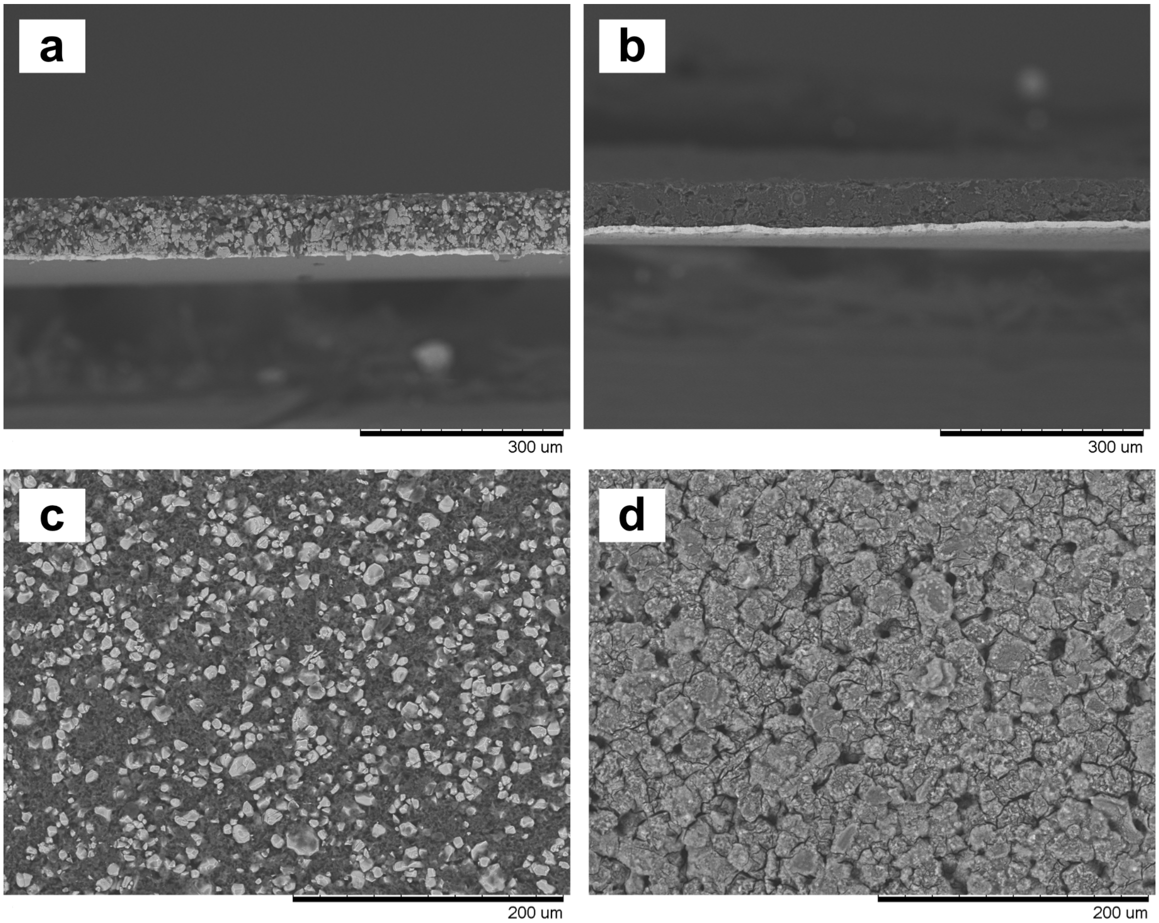

Figure S1. Battery electrodes after 100 charge/discharge cycles and 600 flexing cycles. Cross-sectional SEM micrographs of LCO (a), and graphite (b) electrodes, respectively. Topographical SEM micrographs of LCO (c), and graphite (d) electrodes, respectively.

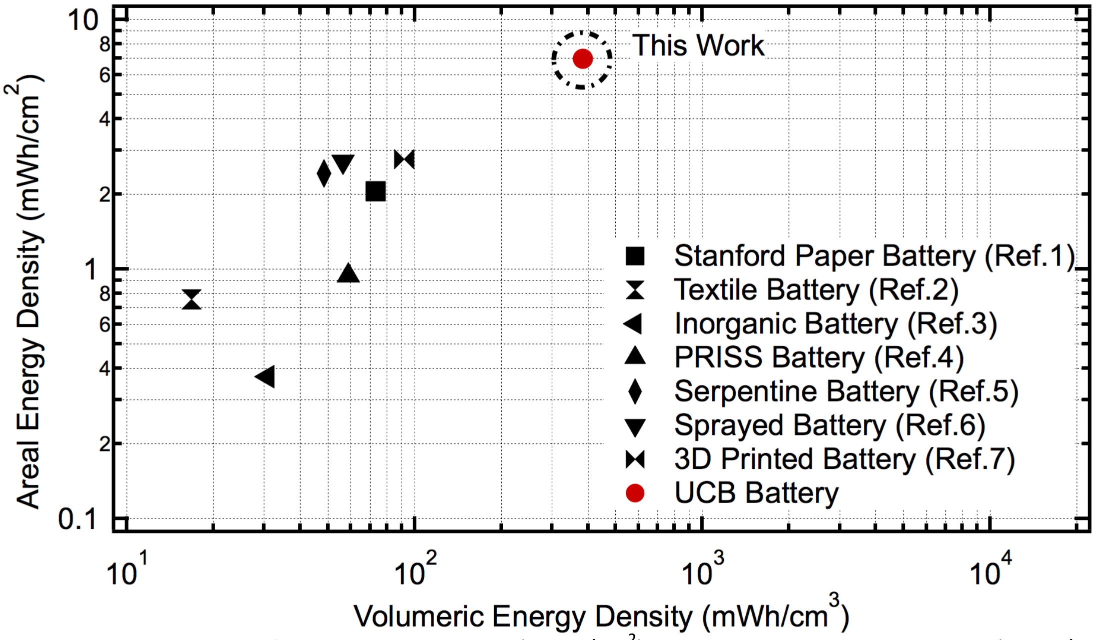

Figure S2. Comparison of areal energy density (mWh/cm^2) and volumetric energy density (mWh/cm^3) of our battery with other flexible batteries based on lithium-ion chemistry reported in the literature. The energy density values are based on unpackaged battery.

Table S1. Comparison of thickness, areal energy density, and volumetric energy density of our battery with other flexible batteries based on lithium-ion chemistry reported in the literature.

	Thickness (μm)*	Areal Energy Density (mWh/cm^2)	Volumetric Energy Density (mWh/cm^3)
**Stanford Paper Battery**[1]	280.0	2.05	73.29
**Textile Battery**[2]	450.0	0.75	16.76
**Inorganic Battery**[3]	6.8	0.37	30.83
**PRISS Battery**[4]	160.0	0.94	58.90
**Serpentine Battery**[5]	500.0	2.42	48.30
**Sprayed Battery**[6]	480.0	2.70	56.25
**3D Battery**[7]	300.0	2.76	92.00
**UCB Battery**	**182.5**	**6.98**	**382.5**

**Thickness of the battery without packaging*

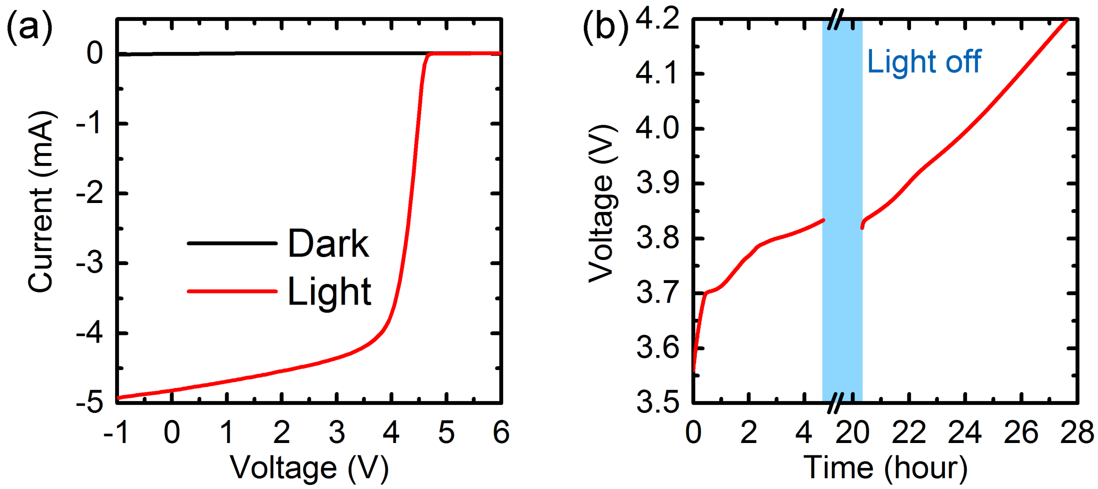

Figure S3. Behavior of PV module and battery with blocking diode. (a) Current-voltage characteristics of PV module with blocking diode in the dark and under 4.8 mW/cm^2 illumination from a compact fluorescent light bulb. (b) Battery charging characteristics under same illumination condition. In the shaded region, the light was turned off but the battery, PV module, and diode remained connected together. The blocking diode prevented current from flowing out of the battery into the PV module.

References

1. Hu, L., Wu, H., La Mantia, F., Yang, Y. & Cui, Y. Thin, flexible secondary Li-ion paper batteries. *ACS Nano* **4,** 5843–5848 (2010).
2. Lee, Y.-H. *et al.* Wearable textile battery rechargeable by solar energy. *Nano Lett.* **13,** 5753–5761 (2013).
3. Koo, M. *et al.* Bendable inorganic thin-film battery for fully flexible electronic systems. *Nano Lett.* **12,** 4810–4816 (2012).
4. Kim, S.-H. *et al.* Printable solid-state lithium-ion batteries: A new route toward shape-conformable power sources with aesthetic versatility for flexible electronics. *Nano Lett.* **15,** 5168–5177 (2015).
5. Xu, S. *et al.* Stretchable batteries with self-similar serpentine interconnects and integrated wireless recharging systems. *Nat. Commun.* **4,** 1543 (2013).
6. Singh, N. *et al.* Paintable battery. *Sci. Rep.* **2,** 481 (2012).
7. Sun, K. *et al.* 3D printing of interdigitated Li-ion microbattery architectures. *Adv. Mater.* **25,** 4539–4543 (2013).
